# Pre-existing Interstitial Lung Abnormalities and Immune Checkpoint Inhibitor-Related Pneumonitis in Solid Tumors: A Retrospective Analysis

**DOI:** 10.1093/oncolo/oyad187

**Published:** 2023-08-17

**Authors:** Kohei Horiuchi, Shinnosuke Ikemura, Takashi Sato, Keitaro Shimozaki, Satoshi Okamori, Yoshitake Yamada, Yoichi Yokoyama, Masahiro Hashimoto, Masahiro Jinzaki, Ikuko Hirai, Takeru Funakoshi, Ryuichi Mizuno, Mototsugu Oya, Kenro Hirata, Yasuo Hamamoto, Hideki Terai, Hiroyuki Yasuda, Ichiro Kawada, Kenzo Soejima, Koichi Fukunaga

**Affiliations:** Division of Pulmonary Medicine, Department of Medicine, Keio University School of Medicine, Tokyo, Japan; Department of Medicine, Icahn School of Medicine at Mount Sinai, Mount Sinai Beth Israel, NY, USA; Division of Pulmonary Medicine, Department of Medicine, Keio University School of Medicine, Tokyo, Japan; Keio Cancer Center, Keio University School of Medicine, Tokyo, Japan; Division of Pulmonary Medicine, Department of Medicine, Keio University School of Medicine, Tokyo, Japan; Department of Respiratory Medicine, Kitasato University School of Medicine, Sagamihara, Japan; Division of Gastroenterology and Hepatology, Department of Internal Medicine, Keio University School of Medicine, Tokyo, Japan; Division of Pulmonary Medicine, Department of Medicine, Keio University School of Medicine, Tokyo, Japan; Department of Radiology, Keio University School of Medicine, Tokyo, Japan; Department of Radiology, Keio University School of Medicine, Tokyo, Japan; Department of Radiology, Keio University School of Medicine, Tokyo, Japan; Department of Radiology, Keio University School of Medicine, Tokyo, Japan; Department of Dermatology, Keio University School of Medicine, Tokyo, Japan; Department of Dermatology, Keio University School of Medicine, Tokyo, Japan; Department of Urology, Keio University School of Medicine, Tokyo, Japan; Department of Urology, Keio University School of Medicine, Tokyo, Japan; Keio Cancer Center, Keio University School of Medicine, Tokyo, Japan; Division of Gastroenterology and Hepatology, Department of Internal Medicine, Keio University School of Medicine, Tokyo, Japan; Keio Cancer Center, Keio University School of Medicine, Tokyo, Japan; Division of Gastroenterology and Hepatology, Department of Internal Medicine, Keio University School of Medicine, Tokyo, Japan; Division of Pulmonary Medicine, Department of Medicine, Keio University School of Medicine, Tokyo, Japan; Division of Pulmonary Medicine, Department of Medicine, Keio University School of Medicine, Tokyo, Japan; Division of Pulmonary Medicine, Department of Medicine, Keio University School of Medicine, Tokyo, Japan; Division of Pulmonary Medicine, Department of Medicine, Keio University School of Medicine, Tokyo, Japan; Clinical and Translational Research Center, Keio University School of Medicine, Tokyo, Japan; Division of Pulmonary Medicine, Department of Medicine, Keio University School of Medicine, Tokyo, Japan

**Keywords:** interstitial lung disease, immune checkpoint inhibitor, lung cancer, renal cell carcinoma, melanoma, gastric cancer

## Abstract

**Background:**

Immune checkpoint inhibitors (ICIs) have demonstrated efficacy over previous cytotoxic chemotherapies in clinical trials among various tumors. Despite their favorable outcomes, they are associated with a unique set of toxicities termed as immune-related adverse events (irAEs). Among the toxicities, ICI-related pneumonitis has poor outcomes with little understanding of its risk factors. This retrospective study aimed to investigate whether pre-existing interstitial lung abnormality (ILA) is a potential risk factor for ICI-related pneumonitis.

**Materials and Methods:**

Patients with non-small cell lung cancer, malignant melanoma, renal cell carcinoma, and gastric cancer, who was administered either nivolumab, pembrolizumab, or atezolizumab between September 2014 and January 2019 were retrospectively reviewed. Information on baseline characteristics, computed tomography findings before administration of ICIs, clinical outcomes, and irAEs were collected from their medical records. Pre-existing ILA was categorized based on previous studies.

**Results:**

Two-hundred-nine patients with a median age of 68 years were included and 23 (11.0%) developed ICI-related pneumonitis. While smoking history and ICI agents were associated with ICI-related pneumonitis (*P* = .005 and .044, respectively), the categories of ILA were not associated with ICI-related pneumonitis (*P* = .428). None of the features of lung abnormalities were also associated with ICI-related pneumonitis. Multivariate logistic analysis indicated that smoking history was the only significant predictor of ICI-related pneumonitis (*P* = .028).

**Conclusion:**

This retrospective study did not demonstrate statistically significant association between pre-existing ILA and ICI-related pneumonitis, nor an association between radiologic features of ILA and ICI-related pneumonitis. Smoking history was independently associated with ICI-related pneumonitis. Further research is warranted for further understanding of the risk factors of ICI-related pneumonitis.

## Teaser

Despite favorable outcomes, immune checkpoint inhibitors are associated with a unique set of toxicities, known as immune-related adverse events (irAEs). Among the toxicities, ICI-related pneumonitis has poor outcomes with little understanding of its risk factors. This retrospective study investigated whether pre-existing interstitial lung abnormality is a potential risk factor for ICI-related pneumonitis.

**Implications for Practice**

This study demonstrated no statistically significant association between pre-existing interstitial lung abnormalities and immune checkpoint inhibitor-related pneumonitis (ICI-related pneumonitis), nor association between radiologic features of interstitial lung abnormalities and ICI-related pneumonitis. Smoking history may be an independent risk factor for ICI-related pneumonitis. While the safety of administering immune checkpoint inhibitors in patients with interstitial lung abnormalities remains to be further investigated, this study suggests that patients with underlying interstitial lung abnormalities may benefit from being administered immune checkpoint inhibitors under careful consideration.

## Introduction

Immune checkpoint inhibitors (ICIs) are approved for use in the treatment of multiple types of cancer and have demonstrated efficacy over previous cytotoxic chemotherapies in clinical trials.^1-7^ Some of the representative agents are anti-programmed cell death protein 1 (PD-1) antibodies such as nivolumab and pembrolizumab, which are used in non-small cell lung cancer (NSCLC), malignant melanoma (MM), renal cell carcinoma (RCC), and gastric cancer (GC). Anti-programmed cell death-ligand 1 (PD-L1) antibody such as atezolizumab is another representative agent commonly used in NSCLC.^8^

Despite their favorable outcomes, they are associated with a unique set of toxicities termed as immune-related adverse events (irAEs). Due to the immunologic nature of the mechanism of these drugs, toxicities typically involve skin, gastrointestinal tract, liver, lungs, endocrine, renal, and nervous systems.^9^ Among the irAEs, ICI-related pneumonitis is a rare but life-threatening toxicity.^10-13^ Incidence was similar in patients with non-small cell lung cancer (NSCLC) and melanoma receiving anti-PD-1/PD-L1 antibody monotherapy (3.3% vs. 3.6%).^14^ While the frequency of grade 3-4 ICI-related pneumonitis was similar across tumor types, the rate of death related to ICI-related pneumonitis was approximately 10% in NSCLC, which is higher than in other cancers.^15-17^ Considering the fatality of ICI-related pneumonitis, understanding the risk factors for ICI-related pneumonitis is critical.^18^

For the past years, pre-existing interstitial lung disease (ILD) was discussed to be a risk factor for ICI-related pneumonitis. However, the results were controversial due to the limited sample sizes in previous studies. In 2 small single-arm trials, nivolumab was safely used in NSCLC with pre-existing mild ILD with an acceptable incidence of ICI-related pneumonitis.^19,20^ To the contrary, one recent single-arm trial administering atezolizumab in NSCLC with pre-existing ILD was terminated due to a high incidence of ICI-related pneumonitis.^21^ In addition, another retrospective study demonstrated that the incidence of ICI-related pneumonitis was significantly higher in patients with pre-existing interstitial lung abnormalities (ILA) defined as increased lung density on lung computed tomography (CT), than those without.^22^ Altogether, whether pre-existing ILA/ILD are potential risk factors for ICI-related pneumonitis remains unclear, which makes clinicians’ decisions to administer ICIs to patients with ILA/ILD difficult in the clinical setting.

To add evidence to this field of interest with uncertainty, we retrospectively examined whether pre-existing ILA, radiologic features of ILA, and other patient characteristics are associated with ICI-related pneumonitis across multiple types of cancer.

## Patients and Methods

### Study Design and Participants

We retrospectively reviewed patients with NSCLC, MM, RCC, and GC, for which ICIs have been approved, who had received anti-PD-1/PD-L1 antibody monotherapy: nivolumab, pembrolizumab, and atezolizumab in clinical practice between September 2014 and January 2019 at Keio University Hospital in Japan. Patients with prior treatment with an anti-CTLA-4 antibody were excluded. The patient population is described in our previous study.^23^ We also excluded patients who did not have prior high-resolution CT (HRCT) (see also Supplementary Material, CT Imaging Protocol) within 1 year of ICI administration. Baseline characteristics, HRCT findings before administration of ICIs, clinical outcomes, and irAEs were collected from medical records. IrAEs were defined as events occurring during PD-1/PD-L1 treatment and events occurring after ICI treatment, including pneumonitis, diarrhea/colitis, hepatitis, rash, neurological disorders, or endocrine abnormalities, diagnosed as being irAEs by attending physicians. IrAEs were graded using the Common Terminology Criteria for Adverse Events version 4.0. The Keio University Hospital Ethics Committee approved this study. Informed consent was obtained in the form of opt-out on the website.

### Assessment of HRCT Findings

**Figure 1.**
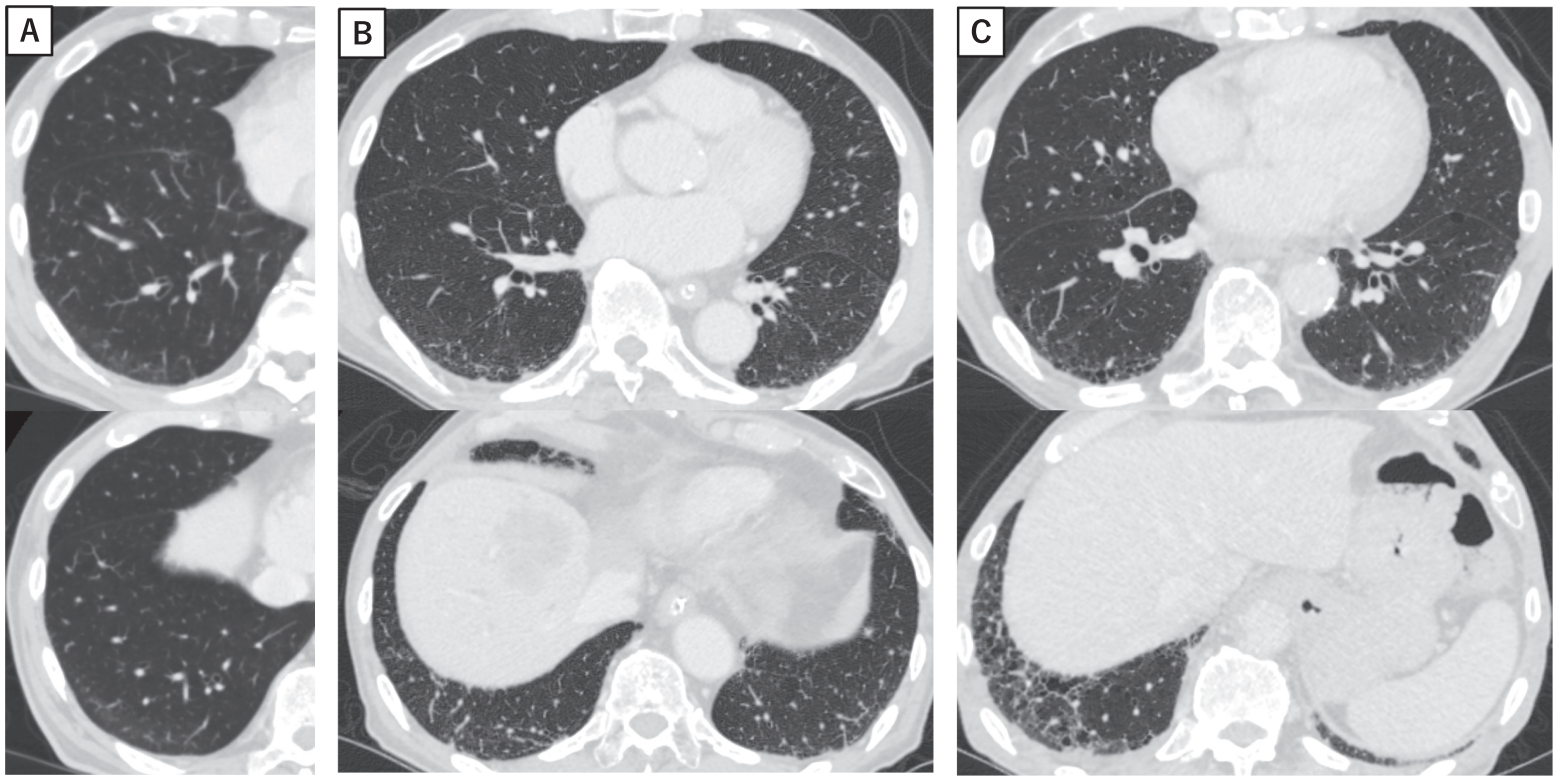
(**A**) Interstitial lung abnormality (ILA) category 1 with ground-glass attenuation (GGA). (**B**) Category 2 with GGA, reticular shadow and traction bronchiectasis. (**C**) Category 3 with GGA, reticular shadow, traction bronchiectasis and honeycombing.

We categorized pre-existing ILA using HRCT within 1 year of ICI administration, along with its radiologic features. ILA was scored from 0 to 3, according to previous studies.^22,24,25^ A score of 0 was defined as no ILA. A score of 1 was defined as focal or unilateral ground-glass attenuation (GGA), focal or unilateral reticulation, or patchy GGA (less than 5% of the lung). A score of 2 was defined as non-dependent GGA affecting more than 5% of any lung zone, non-dependent reticular abnormality, diffuse centrilobular nodularity with GGA, honeycombing, traction bronchiectasis, non-emphysematous cysts, or architectural distortion. A score of 3 was defined as bilateral fibrosis in multiple lobes associated with honeycombing or traction bronchiectasis in a subpleural distribution. A representative image of HRCT is described in Fig. 1.

ILA was scored according to a sequential reading method as previously described.^25^ In the reading process, 1 radiologist and 2 pulmonologists were blinded to clinical data and participated as readers 1, 2, and 3 (Supplementary Fig. S1). Reader 1 reviewed every CT scan and provided a score of 0, 1, 2, or 3. CT scans that were given scores of 1, 2, 3, and 20% of the normal scans (scored 0) were provided to reader 2, who was blinded to the initial interpretation. Scorings that were concordant between readers 1 and 2 were finalized. Reader 3, who was blinded to the interpretations of readers 1 and 2, provided interpretations on the scans discordantly scored between readers 1 and 2. Scorings were finalized for the majority of the scores. Discussions between readers 1, 2, and 3 to reach consensus were required if discordant by all readers.

Once CT scans were scored by the sequential reading method, scores were provided to 2 radiologists. Consensus reading method was performed by them to determine each of the radiologic features: GGA, reticular shadow, honeycombing, diffuse centrilobular nodularity, traction bronchiectasis, and radiation pneumonitis. These radiologic features were classified according to a previous literature.^26^

### Diagnosis of ICI-Related Pneumonitis

The following features were required for the diagnosis of ICI-related pneumonitis: (1) occurring during or after treatment of ICI, (2) new GGA or consolidation preferably on both sides in chest CT, (3) exclusion of pulmonary infection by lack of response to antibiotics or by negative sputum culture, and (4) exclusion of heart failure. Radiologic patterns of ICI-related pneumonitis were classified as usual interstitial pneumonia (UIP) pattern, non-specific interstitial pneumonia (NSIP) pattern, organizing pneumonia (OP) pattern, diffuse alveolar damage (DAD) pattern, or other patterns, according to the American Thoracic Society/European Respiratory Society (ATS/ERS) international multidisciplinary classification of interstitial pneumonias.^27^

### Statistical Analysis

Comparisons between 2 groups were performed using Fisher’s exact test or Mann-Whitney *U* test. Multivariate logistic regression analysis was performed to evaluate risk factors for ICI-related pneumonitis. We selected variables that were found significant in bivariate analyses and preexisting ILA on which we focused in this study. Overall survival (OS) was defined as the time from the initiation of immunotherapy to death from any cause. Kaplan-Meier method was used for survival analysis. The hazard ratios (HRs) and their CIs were estimated using the Cox proportional hazards model. All reported *P*-values were 2-sided and *P*-values <.05 were considered statistically significant. We performed all statistical analyses using EZR version 1.54 and JMP statistical software version 16.2.0.

## Results:

### Patient Characteristics

Among 213 patients with NSCLC, MM, RCC, and GC, who had received anti-PD-1/PD-L1 antibody monotherapies, 209 patients with NSCLC (*n* = 112), MM (*n* = 35), RCC (*n* = 33), and GC (*n* = 29) were reviewed in this study after exclusion of 1 patient with prior treatment with an anti-CTLA-4 antibody and 3 patients who did not have prior CT within 1 year of ICI administration. The median follow-up time was 11.0 months (range, 0.04-53.0) as of July 31, 2019, the analysis cutoff date. The clinical characteristics of enrolled patients are shown in Table 1. The median patient age was 68. Male patients were 144 (68.9%) and Eastern Cooperative Oncology Group (ECOG) performance status (PS) of 0 or 1 was 183 (87.6%). Smoking history was observed in 125 (59.8%) patients and there were prior thoracic radiation in 35 (16.7%) patients. Median duration of ICI administration was 3.79 months (range: 0.04-45.96).

**Table 1.** Patient characteristics.

	All patients (*n* = 209)	Lung cancer patients (*n* = 112)
Age		
Median (range: 25th-75th percentiles)	68 (57-74)	68 (60-75)
Sex		
Male	144	77
Female	65	35
ECOG PS		
0	65	25
1	118	71
2	23	16
3	3	0
Primary tumor		
Lung cancer	112	112
Renal cell carcinoma	33	—
Malignant melanoma	35	—
Gastric cancer	29	—
Smoking history		
Yes	125	80
Never	65	30
Unknown	19	2
Prior radiation		
Thoracic	35	33
Extra-thoracic	33	19
None	141	60
Types of ICIs		
Nivolumab	170	77
Pembrolizumab	33	29
Atezolizumab	6	6
ICI treatment line		
1	37	17
2	72	48
≥3	100	47
Duration of ICI administration		
Median months (range: 25th-75th percentiles)	3.79 (1.50-10.82)	3.38 (1.40-10.12)
Reason for discontinuation of ICIs		
IrAEs	40	25
Progression of disease	121	66
Ongoing ICIs	35	15
Others	13	6
Number of IrAE		
0	102	49
1	65	38
≥2	42	25
Laboratory findings		
Neutrophil-to-lymphocyte ratio		
Median (range: 25th-75th percentiles)	3.33 (2.01-5.33)	3.64 (2.32-6.59)
KL-6 (U/mL)		
Median (range: 25th-75th percentiles)	359 (239-593)	475 (307-778)

Abbreviations: ECOG, Eastern Cooperative Oncology Group; ICI, immune checkpoint inhibitor; IrAE, immune related adverse event; KL-6, Krebs von den lungen-6; PS, performance status.

**Figure 2.**
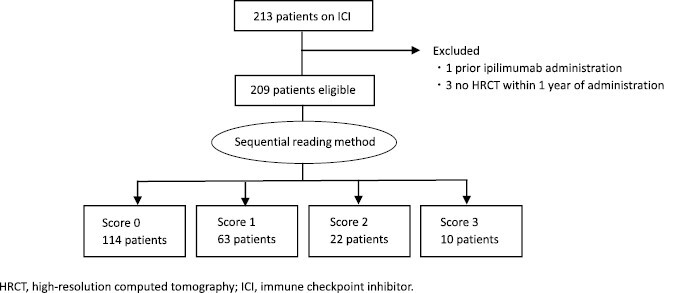
Flow diagram.

In each of 209 patients, CT prior to ICI administration was evaluated. CT prior to ICI administration was within 3 months in 195 patients, 6 months in 11 patients, and 12 months in 3 patients. In all patients, HRCT contiguously covered the entire chest, and section thickness was 1-2mm. A sequential reading method was performed to score ILA from 0 to 3 and none of the CT was discordant by all readers. ILA score was 0 in 114 patients, 1 in 63 patients, 2 in 22 patients, and 3 in 10 patients, respectively. Overall flow diagram is described in Fig. 2 and sequential reading method is described in Supplementary Fig. S1. Subsequently after the scoring of ILA in all patients, consensus reading method was performed by 2 radiologists to evaluate radiologic features. In the study population, the following radiologic features were observed: GGA 89 (42.6%), reticular shadow 68 (32.5%), honeycombing 9 (4.3%), diffuse centrilobular nodularity 3 (1.4%), traction bronchiectasis 29 (13.9%), and radiation pneumonitis 5 (2.4%). The ILA scores for each tumor prior to ICI treatment are summarized in Supplementary Table S1. In patients with malignant melanoma was significantly more common with ILA score negative compared with patients with other tumor types (*P* < .001).

### Incidence and Characteristics of ICI-Related Pneumonitis

The incidence and characteristics of ICI-related pneumonitis among the 209 patients are shown in Table 2. In the study population, 23 (11.0%) patients developed ICI-related pneumonitis, which included 16 patients with lung cancer as the most common tumor group. Line of treatment was 1 or 2 in 15 (65.2%) patients, while 8 (34.8%) patients developed ICI-related pneumonitis at 3 or more lines. Duration of ICI administration was 60 days or more in 15 (65.2%) patients. Pattern of ICI-related pneumonitis was OP in 11 (47.8%) patients, NSIP in 4 (17.4%) patients, DAD in 5 (21.7%) patients, and other patterns in 3 (13.1%) patients. Common grades of ICI-related pneumonitis were 2 and 3 with 16 (69.6%) patients, while 3 had grade 5 events. The steroid was used in 18 (78.3%) patients. Details of patients with ICI-related pneumonitis are described in Supplementary Table S2.

**Table 2.** Incidence and characteristics of ICI-related pneumonitis.

	*n* = 23
Primary tumor	
Lung cancer	16
Renal cell carcinoma	4
Malignant melanoma	2
Gastric cancer	1
Line of treatment	
1	5
2	10
≥3	8
Duration of ICI administration months	
0-59 days	8
60-89 days	3
90 days or more	12
Pattern of ICI-related pneumonitis	
OP	11
NSIP	4
DAD	5
Other	3
CTCAE grade of ICI-related pneumonitis	
1	3
2	7
3	9
4	1
5	3
Corticosteroid use	
Yes	18
No	5

Abbreviations: CTCAE, Common Terminology Criteria for Adverse Events; DAD, diffuse alveolar damage; ICI, immune checkpoint inhibitor; NSIP, nonspecific interstitial pneumonia; OP, organizing pneumonia.

### Comparison of Patient Characteristics in Patients With and Without ICI-Related Pneumonitis

Patient characteristics did not demonstrate a significant difference between patients with and without ICI-related pneumonitis except for smoking history and types of ICIs administered in all patients (Table 3). Smoking history and types of ICIs were significantly associated with ICI-related pneumonitis (*P* = .005 and *P* = .044, respectively). Primary tumor was an increased risk of ICI-related pneumonitis in our study (*P* = .300). Prior radiation was also not associated with an increased risk of ICI-related pneumonitis (*P* = .375). There were no statistically significant differences in the duration of ICI administration (*P* = .539).

**Table 3.** Comparison of patient characteristics between patients with and without ICI-related pneumonitis.

	All patients			Patients with lung cancer		
	ICI-related pneumonitis(+)	ICI-related pneumonitis(−)	*P*-value	ICI-related pneumonitis(+)	ICI-related pneumonitis(−)	*P*-value
	*n* = 23	*n* = 186		*n* = 16	*n* = 96	
Age						
Median (25th-75th percentiles)	68 (59-72)	68 (57-74)	.773	67 (60-73)	68 (60-75)	.577
Sex						
Male	20	124		13	64	
Female	3	62	.056	3	32	.383
ECOG PS						
0	6	59		2	23	
1	15	103		13	58	
2	2	21		1	15	
3	0	3	.853	0	0	.379
Primary tumor						
Lung cancer	16	96		—	—	—
Renal cell carcinoma	4	29				
Malignant melanoma	2	33				
Gastric cancer	1	28	.300			
Smoking history						
Yes	21	104		15	65	
Never	2	63	.005	1	29	.065
Prior radiation						
Thoracic	6	29		6	27	
Extra-thoracic	2	31		0	19	
None	15	126	.375	10	50	.127
Types of ICIs						
Nivolumab	15	155		9	68	
Pembrolizumab	8	25		7	22	
Atezolizumab	0	6	.044	0	6	.192
ICI treatment line						
1	5	32		4	13	
2	10	62		7	41	
≥3	8	92	.391	5	42	.380
Duration of ICI administration						
Median months (25th-75th percentiles)	3.82 (0.77-9.80)	3.75 (1.50-10.74)	.539	2.34 (0.75-4.31)	3.88 (1.50-10.15)	.159
Number of IrAE besides ICI-related pneumonitis						
0	8	102		5	49	
1	7	57		6	33	
≥2	8	27	.052	5	14	.191
Laboratory findings						
Neutrophil-to-lymphocyte ratio						
Median (25th-75th percentiles)	3.89 (2.66-5.61)	3.19 (1.97-5.24)	.128	4.12 (3.31-5.63)	3.52 (2.23-6.60)	.332
KL-6 (U/mL)						
Median (25th-75th percentiles)	315 (247-403)	367 (231-601)	.490	326 (264-529)	490 (329-823)	.055

Abbreviations: ECOG, Eastern Cooperative Oncology Group; ICI, immune checkpoint inhibitor; IrAE, immune related adverse event; KL-6, Krebs von den lungen-6; PS, performance status.

**Figure 3.**
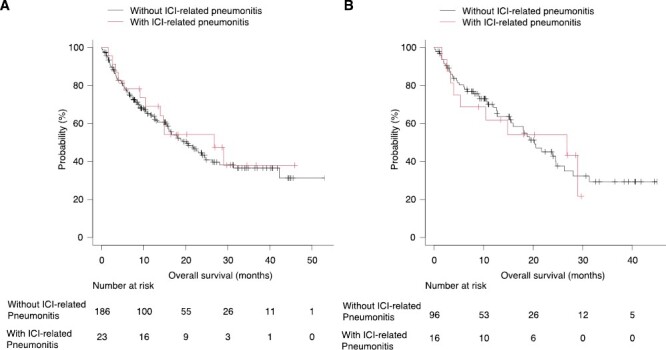
(**A**) Kaplan-Meier describing OS in patients with and without ICI-related pneumonitis, all patients. (**B**) Kaplan-Meier describing OS in patients with and without ICI-related pneumonitis, lung cancer patients.

In all patient group, median OS among patients with and without ICI-related pneumonitis were 26.8 months [(95% CI, 10.4-not available] and 20.3 months [95% CI, 15.9-24.6]) (Fig. 3A). In the univariate analysis, ICI-related pneumonitis was not significantly associated with OS (the HR in patients with ICI-related pneumonitis compared with those without ICI-related pneumonitis was 0.70 [95% CI, 0.37-1.31]). The trend was similar in lung cancer patient group, median OS among patients with and without ICI-related pneumonitis were 26.8 months (95% CI, 4.0-not available) and 20.3 months (95% CI, 15.6-26.3] (Fig. 3B). Again, ICI-related pneumonitis was not significantly associated with OS (the HR in patients with ICI-related pneumonitis compared with those without ICI-related pneumonitis was 0.91 [95% CI, 0.41-2.03]).

### Comparison of Pre-ICI ILA Score in Patients With and Without ICI-Related Pneumonitis

Radiologic findings were compared between patients with and without ICI-related pneumonitis in all patient groups and lung cancer patient groups (Table 4). The scores of ILA (1, 2, and 3) before ICI treatment were not associated with ICI-related pneumonitis in all patient groups (*P* = .428), as well as in patients with lung cancer group (*P* = .570). None of the features of lung abnormalities (GGA, reticular shadow, honeycombing, diffuse centrilobular nodularity, traction bronchiectasis, and radiation pneumonitis) were also associated with ICI-related pneumonitis.

**Table 4.** Comparisons of pre-ICI ILA score between patients with and without ICI-related pneumonitis.

	All patients			Lung cancer patients		
	ICI-related pneumonitis(+)	ICI-related pneumonitis(−)	*p*-value^a^	ICI-related pneumonitis(+)	ICI-related pneumonitis(−)	*P*-value^a^
	*n* = 23	*n* =186		*n* = 16	*n* = 96	
Pre-ICI ILA Score						
0	11	103		8	41	
1	10	53		7	35	
2	1	21		0	12	
3	1	9	.428	1	8	.570
Features of lung abnormalities						
Ground-glass attenuation	10	79	1.000	7	53	.429
Reticular shadow	9	59	.486	5	40	.584
Honeycombing	1	8	1.000	1	7	1.000
Diffuse centrilobular nodularity	0	3	1.000	0	0	NA
Traction bronchiectasis	3	26	1.000	1	19	.296
Radiation pneumonitis	0	5	1.000	0	4	1.000

^a^Fishier’s exact test.

Abbreviations: CT, computed tomography; ICI, immune checkpoint inhibitor; ILA, interstitial lung abnormality; NA, not available.

### Multivariate Logistic Regression Analysis for the Risk Factors of ICI-Related Pneumonitis

In multivariate analysis, smoking history was an independent predictor associated with ICI-related pneumonitis in all patient groups (*P* = .028) (Supplementary Table S3). Types of ICIs and ILA were not found significant as predictors of ICI-related pneumonitis.

### Clinical Course of Score 3 Patients

Details of 10 patients with ILA score of 3 are summarized in Supplementary Table S4. A total of 9/10 (90%) were patients with lung cancer, with a median age of 73.5 (range: 69-90). Honeycomb was confirmed in 9/10 (90%) patients by consensus reading. Baseline pulmonary function tests were performed in 7 patients, as described in the Table. The range of %DLCO was 41.4%-52.5%. At the time of data cutoff, ICI was already terminated due to progressive disease in 9/10 (90%) patients. Median days on ICI was 43 (range:18-459) and one patient developed ICI-related pneumonitis.

## Discussion

In this study, we did not find any statistically significant association between pre-existing ILA and ICI-related pneumonitis, nor association between radiologic features of ILA and ICI-related pneumonitis. To our knowledge, this is the first study to evaluate ILA and specific radiologic features as potential risk factors for ICI-related pneumonitis across multiple types of cancer. In our study, data across multiple types of cancer were collected and integrated because previous literature have not reported a large difference in the prevalence of ICI-related pneumonitis in anti-PD-1/PD-L1 antibody monotherapy across lung cancer and non-lung cancer malignancies.^14^

Based on previous studies, whether pre-existing ILA/ILD is a risk factor for ICI-related pneumonitis has been controversial. In a prospective study, Fujimoto et al reported that 2 out of 18 patients with pre-existing ILDs including 15 UIP patterns that were treated with nivolumab developed ICI-related pneumonitis.^20^ In a retrospective study, Kanai et al investigated 216 patients with NSCLC who were treated with nivolumab. The incidence of ICI-related pneumonitis was 8/26 in patients with pre-existing ILD, which was higher compared with the incidence of 22/190 in patients without pre-existing ILD.^28^ In a retrospective study focusing on pre-existing ILA which has an enlarged definition beyond ILD including increased lung density, Nakanishi et al reported that 6 out of 13 patients with NSCLC with pre-existing ILA that were treated with either nivolumab or pembrolizumab developed ICI-related pneumonitis while the incidence in those without pre-existing ILA was 8 out of 70.^22^ Recently, Ikeda et al reported the result of a phase II trial that investigated the safety of atezolizumab in patients with NSCLC and pre-existing ILD. After enrolling 17 patients (9 UIP pattern), 5 patients developed ICI-related pneumonitis which resulted in the trial termination.^21^ Afterward, it has been considered difficult to carry on further prospective trials regarding this topic. Although there have been more studies reporting an association between pre-existing ILD and ICI-related pneumonitis than those that did not detect the association, the definition of pre-existing ILD investigated in each study differ, and considering a possible publication bias toward studies that demonstrated significance, there has been no clear consensus yet on whether pre-existing ILA or ILD is a risk factor for ICI-related pneumonitis. In light of specific radiologic features, although pre-existing GGA and honeycomb were reported as risk factors for ICI-related pneumonitis,^21,22^ it is still largely unknown whether the risk of ICI-related pneumonitis can be predicted by radiologic features with limited available data.

Our study adds new data to this conflicting area of interest by focusing on ILA through real-world data. ILA is a newly focused definition, applied to detect the early stage of interstitial pulmonary fibrosis (IPF).^29^ This term has evolved to a wide range of usage, and it was termed in our research to define early CT findings of ILD, commonly seen as pre-existing findings in patients at administration of ICI. In clinical practice, determining an the extent of pre-existing ILA or ILD acceptable in administering the use of ICI is a challenge. This study is new in that it may support the use of ICIs, drugs of outstanding survival outcomes, in multiple types of cancer patients with pre-existing ILA under careful consideration.

Honeycomb is one specific radiologic feature typically seen in extensive ILD and IPF, which is discussed as potential risk factor for ICI-related pneumonitis.^21^ Although we did not detect a statistically significant association between pre-existing ILA and ICI-related pneumonitis, it was noteworthy that our data included 9 patients with honeycombing UIP in category 3. Since they had mature follow-up data, we gathered further information and summarized their characteristics and clinical course in Supplementary Table S4. Interestingly, pulmonary function tests were available in 7 patients and the range of %FVC was 83.7%-109.3% and the range of %DLCO was 41.4%-52.5%. In the recently reported trial by Ikeda et al, which was terminated due to a high incidence of ICI-related pneumonitis, the range of %FVC was 80.6%-92.2% and the range of %DLCO was 47.8%-63.3%.^21^ The typical category 3 patients in our study were definable as ILD and had a similar baseline to the terminated trial. However, in our study, ICI-related pneumonitis was reported in only 1 patient in score 3. This indicates the prematurity of the assessment due to the limited sample size in both studies.

As for other risk factors for ICI-related pneumonitis, smoking history was an independent risk factor for ICI-related pneumonitis in our study. This was also pointed out in another retrospective study.^30^ However, its actual causality and mechanism remain unclear. There was a statistically significant difference in the incidence of ICI-related pneumonitis among different ICIs but was not statistically significant under multivariate logistic analyses. This is likely due to the small sample size which resulted in no ICI-related pneumonitis for atezolizumab patients. Prior thoracic radiation was reported as a significant risk factor for ICI-related pneumonitis in a previous study.^31^ However, this was not confirmed in our study. Another study showed that the incidence of ICI-related pneumonitis is higher in lung cancer compared with other tumor types.^32^ This was also not demonstrated in our study. Further investigations are warranted to verify whether and how much these factors can be risks for ICI-related pneumonitis. The small sample size in our study may have contributed to non-significance.

The presence of ICI-related pneumonitis did not have a survival benefit in this study. This is aligned with some of the previous reports demonstrating lower survival benefits of ICI-related pneumonitis among other irAEs such as colitis and dermatitis.^33,34^ While the reason is unclear, some of the reasons may be fewer cases of ICI re-challenge in patients with ICI-related pneumonitis compared with other irAEs.

This study has several limitations. One is the retrospective nature of this study. Prospective studies are warranted for further investigation. Another limitation is that ICI-related pneumonitis was not diagnosed under pathological evaluation. Therefore, there is a small chance that radiologic findings due to other causes were misclassified as ICI-related pneumonitis. Additionally, the number of cases who developed ICI-related pneumonitis, 23 in total, was insufficient to classify and analyze in detail. Therefore, it was not possible to analyze the image patterns of ICI-related pneumonitis in detail, as in previous reports.^35-37^ Lastly, there are technical limitations to manually reviewing CT scans. With the advances in artificial intelligence (AI)-based CT analysis, AI algorithms may have helped classify underlying radiological features more accurately.

## Conclusion

This retrospective study did not demonstrate a statistically significant association between pre-existing ILA and ICI-related pneumonitis, nor association between radiologic features of ILA and ICI-related pneumonitis. Smoking history may be an independent risk factor for ICI-related pneumonitis. While our results may support the use of ICI in patients with ILA, its safety is not yet guaranteed, and further research is warranted for understanding the risk factors of ICI-related pneumonitis.

## Supplementary Material

oyad187_suppl_Supplementary_Tables

oyad187_suppl_Supplementary_Figure_S1

oyad187_suppl_Supplementary_Material

## Data Availability

The data underlying this article will be shared on reasonable request to the corresponding author.
