# Supplementary material for: Pre-existing Interstitial Lung Abnormalities and Immune Checkpoint Inhibitor-Related Pneumonitis in Solid Tumors: A Retrospective Analysis
Source: Oncologist. 2023 Aug 17;29(1):e108–17. doi: 10.1093/oncolo/oyad187 (PMC10769794; doi:10.1093/oncolo/oyad187)
Supplement: oyad187_suppl_Supplementary_Tables [file oyad187_suppl_supplementary_tables.docx]

Supplemental Table 1. Association between tumor types and pre-ICI ILA score

| Tumor type | ILA score 0 | ILA score 1-3 | *p*-value |
| --- | --- | --- | --- |
| Lung cancer | 49 | 63 |  |
| Renal cell carcinoma | 23 | 10 |  |
| Malignant melanoma | 28 | 7 |  |
| Gastric cancer | 14 | 15 | <0.001 |

ILA, interstitial lung abnormalities.

Supplemental Table 2. Details of patients with ICI-related pneumonitis

|  | Patient  1 | Patient  2 | Patient  3 | Patient  4 | Patient  5 | Patient  6 | Patient  7 | Patient  8 | Patient  9 | Patient  10 | Patient  11 | Patient  12 |
| --- | --- | --- | --- | --- | --- | --- | --- | --- | --- | --- | --- | --- |
| Age | 70 | 69 | 62 | 58 | 57 | 78 | 54 | 59 | 73 | 79 | 65 | 68 |
| Sex | F | M | M | M | M | M | F | M | M | M | M | M |
| ECOG PS | 1 | 0 | 1 | 1 | 0 | 1 | 1 | 1 | 1 | 1 | 1 | 2 |
| Primary tumor | Lung cancer | Melanoma | Lung cancer | Lung  cancer | Renal cell  carcinoma | Lung cancer | Lung  cancer | Renal cell  carcinoma | Lung  cancer | Lung cancer | Lung  cancer | Lung  cancer |
| Types of ICI | Nivo | Pembro | Nivo | Pembro | Nivo | Nivo | Pembro | Nivo | Pembro | Nivo | Nivo | Nivo |
| Treatment line | 2 | 1 | 2 | 2 | 3 | 7 | 1 | 2 | 1 | 3 | 2 | 2 |
| Grade of ICI-related pneumonitis  Pattern of ICI-related pneumonitis | 2, OP | 2, OP | 1, OP | 3, OP | 2, OP | 1, Other | 1, NSIP | 3, DAD | 3, Other | 2, OP | 5, DAD | 5, OP |
| Steroid use | None | None | None | Yes | None | None | Yes | Yes | Yes | Yes | Yes | Yes |
| Pre-ICI ILA score | 0 | 1 | 1 | 1 | 0 | 0 | 1 | 0 | 0 | 0 | 1 | 1 |
| Type of ILA |  |  |  |  |  |  |  |  |  |  |  |  |
| GGA | None | Yes | Yes | Yes | None | None | Yes | None | None | None | Yes | None |
| Reticular shadow | None | Yes | None | Yes | None | None | None | None | None | None | Yes | Yes |
| Honeycombing | None | None | None | None | None | None | None | None | None | None | None | None |
| Traction bronchiectasis | None | Yes | None | None | None | None | None | None | None | None | None | None |
|  |  |  |  |  |  |  |  |  |  |  |  |  |
|  | Patient  13 | Patient  14 | Patient  15 | Patient  16 | Patient  17 | Patient  18 | Patient  19 | Patient  20 | Patient  21 | Patient  22 | Patient  23 |  |
| Age | 56 | 69 | 51 | 34 | 73 | 71 | 66 | 60 | 74 | 70 | 78 |  |
| Sex | M | M | M | M | F | M | M | M | M | M | M |  |
| ECOG PS | 0 | 0 | 1 | 1 | 1 | 1 | 1 | 1 | 0 | 0 | 2 |  |
| Primary tumor | Melanoma | Lung  cancer | Lung  cancer | Lung  cancer | Lung  cancer | Renal cell  carcinoma | Lung  cancer | Lung  cancer | Lung  cancer | Renal cell  carcinoma | Gastric  cancer |  |
| Types of ICI | Nivo | Pembro | Nivo | Nivo | Nivo | Nivo | Pembro | Pembro | Pembro | Nivo | Nivo |  |
| Treatment line | 2 | 2 | 3 | 2 | 3 | 2 | 3 | 1 | 1 | 4 | 2 |  |
| Grade of ICI-related pneumonitis  Pattern of ICI-related pneumonitis | 2, NSIP | 5, DAD | 3, DAD | 3, OP | 2, OP | 3, OP | 3, OP | 3, NSIP | 4, DAD | 2, NSIP | 3, Other |  |
| Steroid use | Yes | Yes | Yes | Yes | Yes | Yes | Yes | Yes | Yes | Yes | Yes |  |
| Pre-ICI ILA score | 1 | 3 | 0 | 0 | 0 | 0 | 1 | 1 | 0 | 1 | 2 |  |
| Type of ILA |  |  |  |  |  |  |  |  |  |  |  |  |
| GGA | None | Yes | None | None | None | None | Yes | Yes | None | Yes | Yes |  |
| Reticular shadow | Yes | Yes | None | None | None | None | Yes | None | None | Yes | Yes |  |
| Honeycombing | None | Yes | None | None | None | None | None | None | None | None | None |  |
| Traction bronchiectasis | None | Yes | None | None | None | None | None | None | None | None | Yes |  |

DAD, diffuse alveolar damage; ECOG, Eastern Cooperative Oncology Group; F, female; GGA, ground glass attenuation; ICI, immune checkpoint inhibitor; ILA, interstitial lung abnormality; M, male; Nivo, Nivolumab; NSIP, nonspecific interstitial pneumonia; OP, organizing pneumonia; Pembro, Pembrolizumab; PS, performance status.

Supplemental Table 3. Multivariate logistic analyses

|  | All patients | | |
| --- | --- | --- | --- |
|  | Odds ratio | 95% CI | *p*-value |
| Smoking history | 5.382 | 1.201-24.117 | 0.028 |
| Types of ICIs |  |  | 0.236 |
| Nivolumab | - | - |  |
| Pembrolizumab | 2.369 | 0.876-6.407 | 0.089 |
| Atezolizumab | 0 | 0-NA | 0.999 |
| Pre-existing interstitial lung abnormalities | 1.153 | 0.462-2.876 | 0.761 |

ICI, immune checkpoint inhibitor; CI, confidence interval

Supplemental Table 4. Details of clinical course of patients with score 3 interstitial lung abnormalities

|  | Age | Sex | Primary tumor | Honeycomb | KL-6 (U/mL) | %FVC | %DLCO | ICI | ICI line | Days on ICI | Data cutoff | ICI-ILD |
| --- | --- | --- | --- | --- | --- | --- | --- | --- | --- | --- | --- | --- |
| Patient A | 70 | Male | Lung cancer | Yes | 1626 | NA | NA | Pembrolizumab | 1 | 244 | Ongoing | No |
| Patient B | 90 | Male | Lung cancer | Yes | 443 | 103.3 | 42 | Pembrolizumab | 1 | 168 | PD, terminated | No |
| Patient C | 73 | Female | Lung cancer | Yes | 546 | 83.7 | 44 | Nivolumab | 6 | 125 | PD, terminated | No |
| Patient D | 70 | Male | Lung cancer | Yes | 576 | 88.7 | 52.5 | Nivolumab | 3 | 459 | PD, terminated | No |
| Patient E | 73 | Female | Lung cancer | No | 266 | NA | NA | Nivolumab | 2 | 21 | PD, terminated | No |
| Patient F | 75 | Male | Lung cancer | Yes | 866 | 89.5 | NA | Pembrolizumab | 2 | 44 | PD, terminated | No |
| Patient G | 74 | Male | Gastric cancer | Yes | 910 | 109.3 | NA | Nivolumab | 3 | 42 | PD, terminated | No |
| Patient H | 75 | Male | Lung cancer | Yes | 1979 | 88.9 | 41.4 | Atezolizumab | 2 | 42 | PD, terminated | No |
| Patient I | 69 | Male | Lung cancer | Yes | 1053 | NA | NA | Pembrolizumab | 2 | 21 | PD, terminated | Yes |
| Patient J | 75 | Male | Lung cancer | Yes | 1429 | 91.9 | NA | Nivolumab | 2 | 18 | PD, terminated | No |

DLCO, diffusing capacity of the lungs for carbon monoxide; FVC, forced vital capacity; ICI, immune checkpoint inhibitor; ILD, interstitial lung disease; KL-6, Krebs von den lungen-6; NA, not available; PD, progressive disease.
