# Supplementary figures and images for: Pre-existing Interstitial Lung Abnormalities and Immune Checkpoint Inhibitor-Related Pneumonitis in Solid Tumors: A Retrospective Analysis
Source: Oncologist. 2023 Aug 17;29(1):e108–17. doi: 10.1093/oncolo/oyad187 (PMC10769794; doi:10.1093/oncolo/oyad187)

Supplemental Figure 1. Sequential reading method

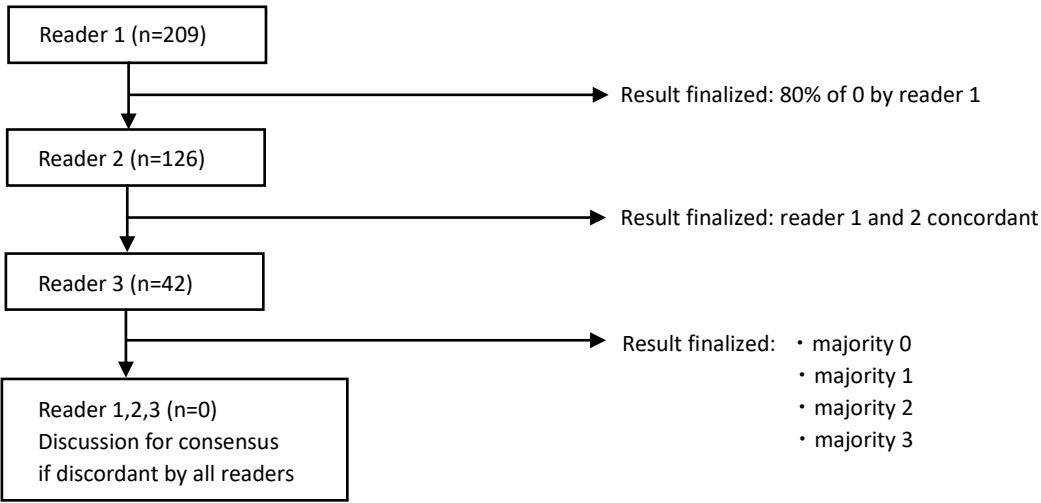

Supplement: oyad187_suppl_Supplementary_Figure_S1 [file oyad187_suppl_supplementary_figure_s1.pdf]
