# Supplementary material for: Pre-existing Interstitial Lung Abnormalities and Immune Checkpoint Inhibitor-Related Pneumonitis in Solid Tumors: A Retrospective Analysis
Source: Oncologist. 2023 Aug 17;29(1):e108–17. doi: 10.1093/oncolo/oyad187 (PMC10769794; doi:10.1093/oncolo/oyad187)
Supplement: oyad187_suppl_Supplementary_Material [file oyad187_suppl_supplementary_material.docx]

**Supplemental material CT Imaging Protocol**

All CT images were obtained during inspiration breath-hold. CT examinations were performed with automatic exposure control (tube current modulation) using a noise index of 10‒15 for a slice thickness of 5 mm. CT scanners were as follows: Aquilion ONE (Canon Medical Systems [former Toshiba Medical Systems], Otawara, Japan), Aquilion 64 (Canon Medical Systems [former Toshiba Medical Systems]), Revolution CT (GE Healthcare, Waukesha, Wisconsin, USA), Revolution EVO EX (GE Healthcare), Discovery CT750HD (GE Healthcare), or BrightSpeed Elite (GE Healthcare). Images with 1 mm slice thickness were reconstructed using standard kernels.
